# Outcomes with frontline immune checkpoint inhibitors among individuals with *BRAF*-mutant non-small cell lung cancer

**DOI:** 10.3389/fonc.2025.1681119

**Published:** 2025-12-10

**Authors:** Julian A. Marin-Acevedo, Ram Thapa, Sudeepthi V. Bandikatla, Dung-Tsa Chen, J. Kevin Hicks, Jhanelle E. Gray, Sonam Puri

**Affiliations:** 1 Thoracic Oncology, Division of Hematology and Medical Oncology, Department of Internal Medicine, Indiana University Melvin and Bren Simon Comprehensive Cancer Center, Indianapolis, IN, United States; 2 Department of Biostatistics, Moffitt Cancer Center, Tampa, FL, United States; 3 Department of Bioinformatics, Moffitt Cancer Center, Tampa, FL, United States; 4 Division of Hematology and Medical Oncology, University of South Florida, Tampa, FL, United States; 5 Division of Hematology and Medical Oncology, Moffitt Cancer Center, Tampa, FL, United States; 6 Division of Individualized Cancer Management, Moffitt Cancer Center, Tampa, FL, United States; 7 Department of Thoracic Oncology, Moffitt Cancer Center, Tampa, FL, United States

**Keywords:** BRAF mutations, NSCLC, adenocarcinoma, smoking, driver mutations, PD-L1, immunotherapy, targeted therapy

## Abstract

**Background:**

Oncogenic *BRAF* mutations affect ~4% of non-small cell lung cancer (NSCLC). Class I mutations (i.e., V600) are typically responsive to BRAF/MEK inhibitors, while non-Class I are often resistant. The role of immune checkpoint inhibitors (ICIs) as first-line therapy in *BRAF*-mutant NSCLC remains unclear.

**Methods:**

We retrospectively analyzed patients with *BRAF*-mutant NSCLC treated at Moffitt Cancer Center from January 1, 2012, to August 9, 2023. Demographics, biomarker (e.g., PD-L1, molecular profile), and treatment data were collected. Progression-free survival (PFS) and overall survival (OS) were estimated using standardized real-world endpoints.

**Results:**

Among 122 individuals, 54 had Class I and 68 had non-Class I mutations. For Class I mutations, ICIs yielded a PFS of 9.2 months and OS of 42.2 months, representing a significant OS advantage over chemotherapy-alone (22.2 months; *p* = 0.03). Outcomes were comparable to those seen with anti-BRAF/MEK therapy (PFS 14.7 months; *p* = 0.49, OS NE; *p* = 0.99), while ICIs trended towards improved OS in those with PD-L1 ≥50% (53.1 vs. 24.8 months; *p* = 0.61). For non-Class I mutations, ICI benefit was more limited (PFS 11.7 months, OS 18.5 months) yet compared favorably to chemotherapy-alone (PFS 4.7 months; *p* = 0.01; OS 9.9 months; *p* = 0.22). No patients with non-Class I mutations received anti-BRAF/MEK therapy.

**Conclusion:**

ICIs appear effective in *BRAF* mutant-NSCLC. For Class I mutations, ICIs yielded significant survival benefit over chemotherapy-alone. Outcomes were comparable to anti-BRAF/MEK therapy, with a potential survival advantage favoring ICIs in those with PD-L1 ≥50%. For non-Class I mutations, ICIs benefit was more modest but compared favorably to chemotherapy-alone.

## 1 Introduction

Oncogenic *BRAF* mutations affect 8% of solid malignancies and 4% of non-small cell lung cancer (NSCLC) (1, 2). *BRAF* mutations upregulate the mitogen-activated protein kinase (MAPK –or– RAS/RAF/MEK/ERK) signaling pathway causing uncontrolled cell proliferation and growth (2, 3). *BRAF* mutations can be categorized into Class I and non-Class I based on the kinase activity, dependency on RAS, and dimerization status (4). Class I mutations (i.e., V600) form potent RAS-independent monomers that can be targeted with BRAF inhibitors. Class II and III mutations, on the other hand, lead to intermediate-potency RAS-independent dimers and low-potency RAS-dependent dimers, respectively (4, 5). BRAF inhibitors have limited activity against BRAF dimerization signaling and are ineffective for most non-Class I mutations (6–8).

Class I and non-Class I *BRAF* mutations occur at similar rates in NSCLC, especially in North America and Europe, but most therapeutic efforts have focused on individuals with Class I mutations (9–11). Anti-BRAF/MEK therapies (i.e., dabrafenib/trametinib or encorafenib/binimetinib) improve survival for Class I *BRAF*-mutant NSCLC and are recommended for frontline use by clinical guidelines (12–14). These recommendations are, however, based on clinical trials that lacked comparator arms for other standard-of-care therapies, and included therapy-naïve and therapy-resistant populations (12–15). Further, the most effective frontline regimens for non-Class I BRAF-mutant NSCLC have not been fully elucidated (16).

Immune checkpoint inhibitors (ICIs) have improved the outcomes for many NSCLC subtypes, but their role in *BRAF*-mutant NSCLC remains poorly defined (17). This is likely a result of the limited representation of these mutations in clinical trials. Retrospective and prospective studies suggest that frontline ICIs improve outcomes, but the study heterogeneity and sampling bias towards Class I mutations, limit definitive conclusions (18–23). We conducted a retrospective analysis to evaluate the role of frontline ICIs in advanced *BRAF*-mutant NSCLC compared to anti-BRAF/MEK agents and chemotherapy, hoping to assist in clinical decision-making and optimize treatment strategies for these patients.

## 2 Materials and methods

### 2.1 Study population

We conducted a retrospective analysis of patients, 18 years of age or older, with NSCLC harboring an oncogenic *BRAF* mutation treated at Moffitt Cancer Center between January 1, 2012, and August 9, 2023. The oncogenic classification of each *BRAF* mutation (i.e., Class I, II, III) was defined based on prior literature and data from the OncoKB™ database (4, 24). Mutations lacking clear oncogenic function or classified as neutral, were categorized as variants of unknown significance (VUS). An internal electronic database containing tumor genomic and pyrosequencing data for all treated patients, identified eligible individuals. Data were collected for individuals with *de-novo* Class I and non-Class I *BRAF* mutations through electronic medical record review. Individuals with incomplete clinical information, acquired *BRAF* mutations (e.g., arising from use of targeted therapies against other oncogenes – *EGFR* or *ALK* –), or those with *BRAF* amplifications or VUS only, were excluded. Demographic information (i.e., sex, self-reported race/ethnicity, tobacco use, age at diagnosis) and disease-specific information (i.e., mutation type, histology, stage, tumor programmed-death ligand 1 [PD-L1] expression, central nervous system [CNS] involvement at presentation) were collected in all eligible individuals. Staging was calculated using the 8^th^ edition of the American Joint Committee on Cancer (AJCC) TNM classification (25). In those with advanced or metastatic disease receiving at least one cycle of palliative systemic therapy, treatment details (i.e., agents, date of initiation and completion, number of cycles, reason for discontinuation) and outcomes datapoints were collected.

This study was conducted with appropriate regulatory measures and was approved by the scientific review committee and the institutional review boards (IRB) at Moffitt Cancer Center (Approval: MCC 19335; August 14, 2023). Informed consent was waived by the IRB due to the retrospective nature of the analysis.

### 2.2 Genomic profiling and PD-L1 testing

Genomic data were retrieved from the internal database containing results from different platforms including immunohistochemistry, next-generation sequencing (NGS) assays from both commercial and in-house platforms (e.g., liquid-based [FoundationOne Liquid CDx, Guardant] and tissue-based tests [FoundationOne CDx, Moffitt STAR]), pyrosequencing (Moffitt Pyrosequencing), mass spectrometry-based assays (MassARRAY), and single nucleotide polymorphism (SNP) genotyping (SNaPshot).

PD-L1 expression was assessed using the recommended procedure for commercially available IHC tests VENTANA PD-L1 (SP263) and PD-L1 IHC 22C3 PharmDx (26). PD-L1 was reported as tumor proportion score (TPS). Positivity was defined as TPS ≥1%, and high expression as ≥50%.

### 2.3 Outcomes

Outcomes analysis was pursued in those with advanced/metastatic disease receiving at least one cycle of either ICI-based therapy, anti-BRAF/MEK therapy, or chemotherapy-alone. Individuals treated under clinical trials or treated with other systemic therapies were excluded from outcomes analysis. Treatment response was assessed by the investigators and defined as the best documented clinical and/or radiographic response reported in the medical record. Responses were categorized using standardized real-world endpoints (27, 28). Complete response (CR) was defined as a reported complete resolution of disease by the treating clinician or radiographic report. Partial response (PR) was defined as a documented partial reduction in the disease burden or clinical improvement. Stable disease (SD) was defined as no significant clinical or radiographical change. Progressive disease (PD) was defined as an increase in disease burden, appearance of new lesions, or clinical progression deemed by the treating physician. Real-world progression-free survival (PFS) was defined as the time from initiation of first-line systemic therapy until PD, death, or lost to follow-up. Real-world overall survival (OS) was defined as the time from initiation of first-line systemic therapy until death from any cause or end of follow-up. Those without PD, death, or lost to follow-up were censored at the most recent encounter.

### 2.4 Statistical analysis

Descriptive statistics were used to summarize the data. For continuous variables, mean, median, and ranges were reported. Comparisons between groups were made using either *t*-test or non-parametric Mann-Whitney *U* test. Categorical variables were summarized using frequencies and proportions. Group comparisons were made using Chi-squared test or Fisher’s exact test. PFS and OS were estimated using the Kaplan-Meier method, and subgroups differences were assessed through log-rank test. Cox proportional hazards regression was used to estimate 95% confidence intervals (CIs). A *p* value of ≤0.05 was defined as significant. All statistical analyses were conducted using R Statistical Software version 4.4.2.

## 3 Results

We identified 122 patients with *BRAF*-mutant NSCLC: 54 Class I, 37 Class II, and 31 Class III mutations (**Table 1**). The median age was 67 years (range 40 – 91). Those with *BRAF* Class I NSCLC were slightly younger than non-Class I (65 vs. 69 years; *p* = 0.02). Females made up 52% of the entire cohort, while most males (60%) had non-Class I mutations. Up to 88% of patients self-identified as White. Over 95% had adenocarcinoma and 85% had at least stage III disease at presentation. CNS involvement was present at diagnosis in 21% of individuals with stage IV disease (17/81), the majority of whom harbored non-Class I mutations (12/17; 71%). History of tobacco use was common (81%), but never-smokers were disproportionately represented among Class I mutations (61%).

**Table 1:** Demographics and clinical variables.

	All n=122	Class I n=54	non-Class I n=68 [37 Class II, 31 Class III]	*P value*
Median Age, years (range)	66.8 (40 – 91)	64.6 (44 – 84)	68.6 (40 – 91)	**0.02**
Sex				
Female	63 (51.6%)	30 (55.6%)	33 (48.5%)	0.56
Male	59 (48.4%)	24 (44.4%)	35 (51.5%)	
Race/ethnicity				
White	107 (87.7%)	49 (90.7%)	58 (85.3%)	0.35
Hispanic	7 (5.7%)	1 (1.9%)	6 (8.8%)	
Black	5 (4.1%)	3 (5.6%)	2 (2.9%)	
Asian	3 (2.5%)	1 (1.9%)	2 (2.9%)	
Tobacco use history				
Yes	99 (81.1%)	40 (74.1%)	59 (86.8%)	0.12
No	23 (18.9%)	14 (25.9%)	9 (13.2%)	
Histology				
Adenocarcinoma	116 (95.1%)	51(94.4%)	65 (95.6%)	0.83
Squamous cell	3 (2.5%)	1 (1.9%)	2 (2.9%)	
Other	3 (2.5%)	2 (3.7%)	1 (1.5%)	
Stage at diagnosis (AJCC 8^th^ Edition)				
I	14 (11.5%)	6 (11.1%)	8 (11.8%)	0.22
II	4 (3.3%)	0 (0.0%)	4 (6.3%)	
III	23 (18.9%)	13 (24.1%)	10 (14.7%)	
IV	81 (66.4%)	35 (64.8%)	46 (67.6%)	
CNS metastasis at diagnosis*				
Yes	17 (13.9%) [14.4%]	5 (9.3%) [9.6%]	12 (17.6%) [18.2%]	0.29
No	101 (82.3%) [85.6%]	47 (87.0%) [90.4%]	54 (79.4%) [81.8%]	
Unknown	4 (3.3%)	2 (3.7%)	2 (2.9%)	
PD-L1*				
<1%	18 (14.8%) [25.7%]	1 (1.9%) [4.3%]	17 (25.0%) [36.2%]	**<0.01**
1-49%	11 (9.0%) [15.7%]	6 (11.1%) [26.1%]	5 (7.4%) [10.6%]	
≥50%	41 (33.6%) [58.6%]	16 (29.6%) [69.6%]	25 (36.8%) [53.2%]	
Unknown	52 (42.6%)	31 (57.4%)	21 (38.9%)	
First-line therapy for advanced disease				
Anti-BRAF	15 (12.3%)	15 (27.8%)	0 (0.0%)	–
ICI	25 (20.5%)	7 (13.0%)	18 (26.5%)	
Chemo-ICI	31 (25.4%)	7 (13.0%)	24 (35.3%)	
Chemotherapy	22 (18.0%)	11 (20.4%)	11 (16.2%)	
Other	6 (4.9%)	1 (1.9%)	5 (7.4%)	
None	23 (18.9%)	13 (24.1%)	10 (14.7%)	

AJCC, American Joint Committee on Cancer; CI, Confidence Interval); CNS, Central Nervous System; ICI, Immune Checkpoint Inhibitors; mo, Months; NE, Non-estimable; OS, Overall Survival; PFS, Progression-Free Survival.

*Percentages in parentheses are calculated based on the total number of patients in each column. Percentages in square brackets represent the proportion among patients with available data (applies to PD-L1 and CNS metastasis variables). Highlight statistical significance.

### 3.1 PD-L1 levels

There were 70 patients with PD-L1 testing available of which 52 (74%) had a TPS ≥1%. Those with Class I mutations were more likely to have PD-L1 expression ≥1% (96% vs. 64%) and ≥50% (70% vs. 53%) compared to those with non-Class I mutations (*p* < 0.01).

### 3.2 Molecular landscape

*BRAF* mutations were detected on NGS for most patients (81%), while other detection methods (i.e., pyrosequencing, genotyping) were less common (**Supplementary Table 1S****).** Because NGS platforms identified specific *BRAF* variants and other co-mutations, the mutational landscape analysis was limited to individuals with available NGS data.

**Figure 1:**
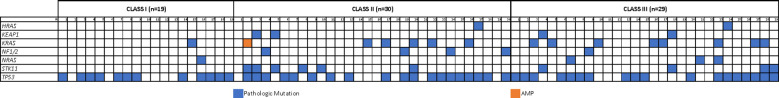
Mutational landscape. OncoMap showing the genomic distribution for different co-mutations according to *BRAF* mutation class.

In those with Class I mutations on NGS, all had a V600E variant (n=32) and 63% (n=19) harbored additional pathogenic mutations. *TP53* was the most common (n=12/19; 63%) while other co-mutations were rare (**Figure 1**, **Supplementary Figure 1S**). In patients with non-Class I mutations on NGS, the most common *BRAF* variants were G469X (n=18; 28%), N581X (n=8; 12%), D594X (n=8; 12%), G466X (n=7; 11%), and K601X (n=6; 9%) (**Supplementary Table 2S**). Among these individuals, 91% (n=59) had additional pathogenic mutations, including *TP53* (n=37/59; 63%), *RAS* (n=19/59; 32% - *KRAS* 24%, *NRAS* 5%, *HRAS* 3%), *STK11* (n=10/59; 17%), *NF1* (n=5/59; 8%), and *KEAP1* (n=4/59; 7%) (**Figure 1**, **Supplementary Figure 1S**). While concurrent *EGFR* alterations were identified in a small subset of patients with Class I (n=5) and non-Class I (n=9) *BRAF* mutations, only a minority harbored sensitizing variants and received anti-EGFR therapy, suggesting that most *EGFR* co-mutations were not clinically actionable (**Supplementary Table 3S**).

### 3.3 Clinical outcomes: real world progression-free survival and overall survival

**Figure 2:**
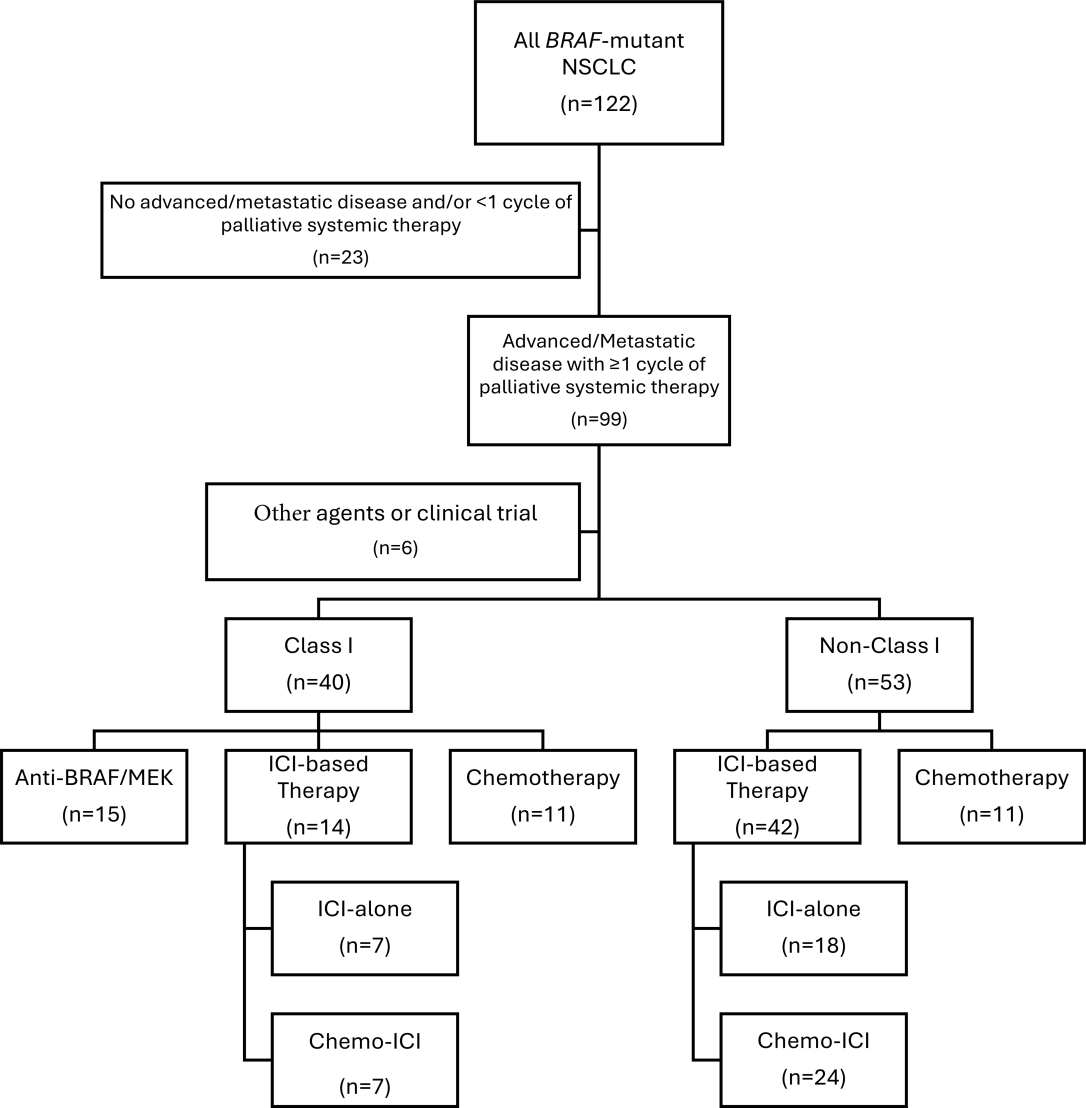
Diagram of evaluable population. CONSORT Flow Diagram depicting individuals eligible for outcomes evaluation according to therapy. ICI, immune checkpoint inhibitor; NSCLC, non-small cell lung cancer.

Among the 122 patients, 99 had advanced/metastatic disease treated with at least one cycle of palliative systemic therapy. Of these, 56 received frontline ICI-based therapies including 25 ICI-alone (7 Class I, 7 Class II, 11 Class III) and 31 chemo-ICI (7 Class I, 12 Class II, 12 Class III). Fifteen patients received anti-BRAF/MEK therapy (all Class I) and 22 received chemotherapy-alone (11 Class I, 6 Class II, 5 Class III). Six patients (1 Class I, 4 Class II, 1 Class III) were treated with clinical trial or other targeted agents and were excluded from the outcomes analysis (**Figure 2****) (****Supplementary Table 3S**).

**Figure 3:**
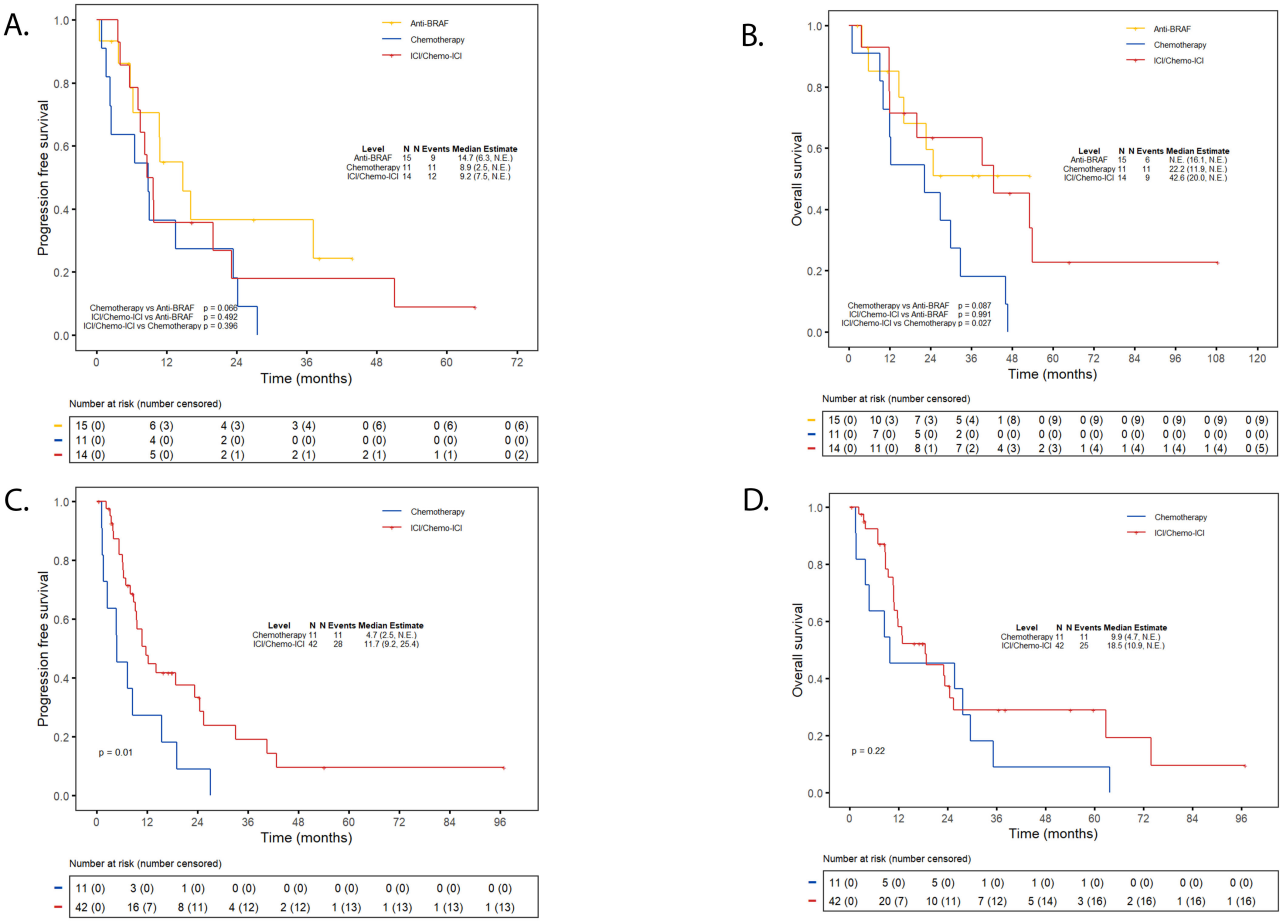
Progression-free survival and overall survival first-line immunotherapy. **(A)** PFS ICI/chemo-ICI vs. targeted therapy or chemotherapy in class (I) **(B)** OS ICI/chemo-ICI vs. targeted therapy or chemotherapy in class (I) **(C)** PFS ICI/chemo-ICI vs. chemotherapy in non-class (I) **(D)** OS ICI/chemo-ICI vs. chemotherapy in non-class (I) Kaplan Meier Curves depicting progression-free survival (PFS) for individuals with class I *BRAF* mutations treated with frontline ICI-based therapy compared to anti-BRAF/MEK therapy or chemotherapy-alone **(A)** Overall survival (OS) for individuals with class I treated with frontline ICI-based therapy compared to anti-BRAF/MEK therapy or chemotherapy-alone **(B)** PFS for individuals with non-class I mutations treated with frontline ICI-based therapy compared to chemotherapy-alone **(C)** OS for individuals with non-class I mutations treated with frontline ICI-based therapy compared to chemotherapy-alone **(D)** ICI, immune checkpoint inhibitor; NE, Not Estimable.

In patients with Class I mutations, the efficacy of ICI-based therapies was comparable to anti-BRAF/MEK therapy and conferred a survival advantage over chemotherapy. Specifically, the median PFS with ICI/chemo-ICI was similar to chemotherapy (9.2 vs. 8.9 months; *p* = 0.40) and not significantly different than anti-BRAF/MEK therapy (14.7 months; *p* = 0.49) (**Figure 3A**). However, the median OS was significantly longer with ICI/chemo-ICI than with chemotherapy (42.6 vs. 22.2 months; *p* = 0.03) and not significantly different compared to anti-BRAF/MEK therapy (not estimable [NE]; *p=*0.99) (**Figure 3B**). In those treated with frontline ICI/chemo-ICI, subsequent treatments included anti-BRAF/MEK inhibitors (43%; n=6/14), other ICI-based regimens (14%; n=2/14), or chemotherapy (14%; n=2/14). For those treated with frontline anti-BRAF/MEK therapy, subsequent therapies included ICI-based treatments (15%; n=3/15) and chemotherapy (7%; n=1/15), while 60% (n=9/15) received no additional treatments. In the chemotherapy group, 27% (n=3/11) received additional chemotherapy, 27% (n=3/11) received ICI-based therapy, 9% (n=1/11) received anti-BRAF/MEK therapy, 9% (n=1/11) were enrolled in clinical trials, and 27% (n=3/11) received no additional therapy (**Supplementary Table 4S**).

For non-Class I patients, treatment with ICI/chemo-ICI was associated with a significantly better PFS compared to chemotherapy (11.7 vs. 4.7 months; *p* = 0.01), and a non-significant trend toward improved OS (18.5 vs. 9.9 months; *p* = 0.22) (**Figures 3C, D**). For those treated with frontline ICI/chemo-ICI, subsequent therapies included anti-BRAF/MEK therapy (5%; n=2/42), a different ICI-based treatment (7%; n=3/42), and chemotherapy (14%; n=6/42) while the majority (67%; n=28/42) received no additional treatment. Subsequent therapies for those treated with frontline chemotherapy included additional chemotherapy (9%, n=1/11), ICI-based therapy (45%; n=5/11), and 36% (n=4/11) received no further therapy (**Supplementary Table 4S**).

We found no significant differences in outcomes between individuals with Class I and non-Class I treated with ICI/chemo-ICI or chemotherapy (**Supplementary Figure 2S****).**

### 3.4 Exploratory subgroup analyses

#### 3.4.1 PD-L1 levels

For individuals with Class I mutations and PD-L1 ≥1% (n=21), both ICI-based and anti-BRAF/MEK therapies yielded comparable outcomes (**Table 2**). There was a trend towards improved OS with higher PD-L1 levels in those treated with ICI-based therapy. This was particularly striking for individuals with TPS ≥50% (n=7) (53.1 vs. 24.8 months; p=0.61). While no statistical significance was achieved, this finding suggests that individuals with Class I mutations and high PD-L1 expression could derive clinically relevant long-term benefit from ICI-based therapy. Only 1 patient with Class I mutation had PD-L1 <1%, thus no formal analysis was pursued between treatment arms.

**Table 2:** Outcomes by PD-L1 levels, tobacco use, and CNS metastasis.

			ICI-based [n=56; 14 Class I, 42 non-Class I]	Chemotherapy [n=22; 11 Class I, 11 non-Class I]	ICI-based vs. Chemotherapy *P*	Anti-BRAF/MEK [n=15; all Class I]	ICI-based vs. Anti-BRAF/MEK *P*	Chemotherapy vs. Anti-BRAF/MEK *P*
Class I								
OS (mo) (95% CI)	Overall		42.6 (20.0 – NE)	22.2 (11.9 – NE)	**0.03**	NE (16.1 – NE)	0.99	0.09
	PD-L1%	<1	N/A [0]	N/A [0]	–	38.2 [1]	–	–
		≥1	39.2 (20.0 – NE) [11]	N/A [0]	–	24.8 (22.7 – NE) [10]	0.94	–
		1-49	25.5 (11.8 – NE) [4]	N/A [0]	–	14.7 (14.7 – NE) [2]	0.45	–
		≥50	53.1 (20.0 – NE) [7]	N/A [0]	–	24.8 (22.7 – NE) [8]	0.61	–
	Tobacco use	Yes	36.5 (12.0 – NE) [8]	19.6 (10.1 – NE) [8]	0.14	NE (16.1 – NE) [11]	0.86	0.22
		No	42.6 (39.2 – NE) [6]	22.2 (11.9 - NE) [3]	0.62	24.8 (14.7 – NE) [4]	1.00	0.31
	CNS	Yes	11.8 [1]	17.0 (9.1 – NE) [4]	–	–	–	–
		No	42.6 (20.0 – NE) [13]	26.9 (9.1 – NE) [7]	**0.03**	NE (16.1 – NE) [15]	0.82	0.14
PFS (mo) (95% CI)	Overall		9.2 (7.5 – NE)	8.9 (2.5 – NE)	0.40	14.7 (6.3 – NE)	0.49	0.07
	PD-L1%	<1	N/A [0]	N/A [0]	–	38.2 [1]	–	–
		≥1	9.7 (7.5 – NE) [11]	N/A [0]	–	10.9 (6.3 – NE) [10]	0.74	–
		1-49	7.1 (4.1 – NE) [4]	N/A [0]	–	12.8 (10.9 – NE) [2]	0.71	–
		≥50	9.8 (7.5 – NE) [7]	N/A [0]	–	10.8 (5.8 – NE) [8]	0.97	–
	Tobacco use	Yes	14.9 (8.3 – NE) [8]	4.5 (2.3 – NE) [8]	0.14	16.1 (5.8 – NE) [11]	0.90	**0.05**
		No	7.9 (5.6 – NE) [6]	13.5 (9.0 – NE) [3]	0.25	12.8 (6.3 – NE) [4]	0.35	0.78
	CNS	Yes	4.1 [1]	11.3 (6.1 – NE) [4]	–	N/A [0]	–	–
		No	9.7 (7.5 – NE) [13]	2.5 (1.7 – NE) [7]	0.25	14.7 (6.3 – NE) [15]	0.61	0.06
Non-class I								
OS (mo) (95% CI)	Overall		18.5 (10.9 – NE)	9.9 (4.7 – NE)	0.22			
	PD-L1%	<1	18.5 (12.8 – NE) [12]	16.7 (1.5 – NE) [4]	0.81			
		≥1%	18.8 (10.8 – NE) [22]	25.7 (9.9 – NE) [3]	0.60			
		1-49	9.8 (7.0 – NE) [4]	25.7 [1]	–			
		≥50	23.1 (10.9 – NE) [17]	36.8 (9.9 -NE) [2]	0.63			
	Tobacco use	Yes	18.8 (11.9) [36]	9.9 (4.7 – NE) [11]	0.26			
		No	9.7 (3.5 – NE) [6]	N/A [0]	–			
	CNS	Yes	12.9 (10.8 – NE) [7]	N/A [0]	–			
		No	23.1 (10.9 – NE) [35]	17.8 (4.7 – NE) [10]	0.24			
PFS (mo) (95% CI)	Overall		11.7 (9.2 – 25.4)	4.7 (2.5 – NE)	**0.01**			
	PD-L1%	<1	14.2 (6.2 – NE) [12]	3.0 (1.2 – NE) [4]	0.08			
		≥1	10.8 (8.8 – NE) [22]	15.5 (7.4 – NE) [3]	0.49			
		1-49	9.4 (4.0 – NE) [4]	15.5 [1]	–			
		≥50	18.8 (9.2 – NE) [17]	17.2 (7.4 – NE) [2]	0.63			
	Tobacco use	Yes	14.2 (9.4 – 33.0) [36]	4.7 (2.5 – NE) [11]	**<0.01**			
		No	7.5 (3.5 – NE) [6]	N/A [0]	–			
	CNS	Yes	10.8 (9.2 – NE) [7]	N/A [0]	–			
		No	11.7 (8.8 – 33.0) [35]	6.1 (2.5 – NE) [10]	**0.02**			

CI, Confidence Interval; CNS, Central Nervous System; ICI, Immune Checkpoint Inhibitors; N/A, Not Applicable; NE, Not Estimable. Highlight statistical significance.

The sample size was limited for patients with non-Class I mutations and PD-L1 ≥1% (n=25), thus no meaningful differences were seen between ICI-based therapies and chemotherapy. Specifically, individuals with PD-L1 ≥50% treated with ICI/chemo-ICI (n=17) or chemotherapy (n=2) had similar PFS (18.8 vs. 17.2 months; *p* = 0.63) and OS (23.1 vs. 36.8 months; *p* = 0.63). In contrast, patients with PD-L1 <1% (n=12) had surprisingly longer PFS (14.2 vs. 3.0 months; *p* = 0.08) and OS (18.5 vs. 16.7 months; *p* = 0.81) compared to chemotherapy (n=4). While this could suggest that ICI-based therapy could be more effective in this subgroup, the number of patients limits definitive conclusions.

#### 3.4.2 ICI-alone and chemo-ICI

An analysis among those treated with ICI-based therapies demonstrated that ICI-alone (n=25) was associated with significantly improved PFS (10.8 vs. 7.0 months; *p* < 0.01) and OS (23.1 vs. 17.2 months; *p* = 0.04) compared to chemo-ICI (n=31). This difference is likely confounded by imbalances in PD-L1 expression between the groups. Up to 89% (n=16/18) of patients receiving ICI-alone had PD-L1 ≥50% compared to 30% (n=8/27) in the chemo-ICI group.

For patients with Class I mutations, outcomes with ICI-alone (n=7) or chemo-ICI (n=7) were comparable to anti-BRAF/MEK therapy (n=15) and trended toward improved OS relative to chemotherapy (n=11) (**Table 3**, **Supplementary Figure 3S**). Conversely, in patients with non-Class I mutations ICI-alone was associated with greater benefit than chemo-ICI. Specifically, ICI-alone (n=18) led to a significant improvement in PFS compared to chemotherapy (n=11) (18.8 vs. 4.7 months; *p* < 0.01). This was not seen with chemo-ICI (n=24) (10.8 months; *p=*0.09) (**Supplementary Figure 3S**). This finding could also have resulted from the higher PD-L1 expression within the ICI-alone versus the chemo-ICI subgroup (PD-L1 ≥50%: 93% [n=13/14] vs. 20% [n=4/20], respectively).

**Table 3:** Outcomes with ICI-alone or chemo-ICI compared to chemotherapy-only and anti-BRAF/MEK therapy.

	ICI-Alone (n=25; 7 Class I, 18 non-Class I)	Chemo-ICI (n=31; 7 Class I, 24 non-Class I)	Chemotherapy (n=22; 11 Class I, 11 non-Class I)	ICI vs. Chemotherapy *P*	Chemo-ICI vs. Chemotherapy *P*	Anti-BRAF/MEK (n=15)	ICI vs. Anti-BRAF/MEK *P*	Chemo-ICI vs. Anti-BRAF/MEK *P*
OS (mo) (95% CI)	23.1 (11.9 – NE)	20.0 (11.8 - 53.9)	17.2 (9.9 – 29.8)	**0.04**	0.15			
Class I	42.6 (11.8 – NE)	39.2 (20.0 – NE)	22.2 (11.9 – NE)	0.10	0.06	NE (16.1 – NE)	0.80	0.91
Non-Class I	23.1 (10.9 – NE)	12.9 (10.7 – NE)	9.9 (4.7 – NE)	0.22	0.34			
PFS (mo) (95% CI)	10.8 (8.3 – NE)	9.8 (8.0 – 23.2)	7.0 (2.5 – 15.5)	**<0.01**	0.10			
Class I	8.3 (5.6 – NE)	9.8 (7.1 – NE)	8.9 (2.5 – NE)	0.38	0.89	14.7 (6.3 – NE)	0.64	0.42
Non-Class I	18.8 (9.4 – NE)	10.8 (8.0 – 33.0)	4.7 (2.5 – NE)	**<0.01**	0.09			

CI, Confidence Interval; ICI, Immune Checkpoint Inhibitors; N/A, Not applicable; NE, Not Estimable. Highlight statistical significance.

#### 3.4.3 Tobacco use

Tobacco use seemed to influence outcomes in patients treated with ICI-based therapies (**Table 2**). Never-smokers (n=12) demonstrated significantly shorter PFS compared to tobacco users (n=44) in the overall cohort (7.9 vs. 14.2 months; *p* = 0.04, **Supplementary Figure 4S**). This trend persisted within the Class I subgroup (7.9 vs. 14.9 months; *p* = 0.14). In contrast, OS was numerically more favorable among never-smokers, both in the overall cohort (39.2 vs. 20.0 months; *p* = 0.84, **Supplementary Figure 4S**) and within the Class I subgroup (42.6 vs. 36.5 months; *p* = 0.90). Never-smokers with Class I mutations (n=6) also trended towards improved OS with ICI/chemo-ICI compared to anti-BRAF/MEK therapy (n=4) (24.8 months; *p* = 1.00) or chemotherapy (n=3) (22.2 months; *p* = 0.62). These differences, however, did not reach statistical significance likely due to the limited cohort size.

In patients with non-Class I mutations, tobacco users treated with ICI-based therapies (n=36) showed a trend towards improved PFS (14.2 vs. 7.5 months; *p* = 0.07) and OS (18.8 vs. 9.7 months; *p* = 0.07) compared to never-smokers (n=6). Tobacco users also had significantly better PFS (14.2 vs. 4.7 months; p<0.01), and numerically higher OS (18.8 vs. 9.9 months; *p* = 0.26) compared to those treated with chemotherapy (n=11).

#### 3.4.4 CNS involvement

Given the limited number of patients with CNS involvement, formal comparisons across treatment arms were not feasible. In those treated with ICI-based therapies (n=8), however, OS was significantly shorter for patients with CNS involvement (12.4 months) than without CNS involvement (24.6 months; *p* = 0.05; **Supplementary Figure 5S**, **Supplementary Table 5S**). In patients without CNS involvement, ICI-based therapies were particularly beneficial for individuals with Class I mutations (n=13). PFS and OS in this subgroup (9.7 and 42.6 months, respectively) were comparable to those observed with anti-BRAF/MEK therapy (n=15) (PFS 14.7 months, *p* = 0.61; OS NE, *p* = 0.86) while OS was significantly improved compared to chemotherapy (n=7) (26.9 months; *p* = 0.03). In those with non-Class I mutations and no CNS involvement, the benefit of ICI/chemo-ICI (n=35) was less pronounced. PFS however, remained significantly longer compared to chemotherapy (n=10) (11.7 vs. 6.1 months; p=0.02) and OS showed a non-significant trend toward improvement (23.1 vs. 17.8 months; p=0.24) (**Table 2**).

## 4 Discussion

This study represents one of the largest single-institution analyses to-date evaluating the role of frontline ICI-containing regimens across all subclasses of *BRAF*-mutant NSCLC in North America. Our findings support previous reports where individuals with *BRAF*-mutant NSCLC derived meaningful clinical benefit from ICI-based regimens (20, 22, 23, 29).

For individuals with Class I mutations, current guidelines recommend frontline anti-BRAF/MEK therapy based on the survival benefit seen in clinical trials (12–14). These therapies, however, are associated with substantial toxicities (i.e., rash, fever, diarrhea, reduced ejection fraction, retinopathy) that may limit their use and impact quality of life. Further, individuals with melanoma and Class I mutations experience better survival with frontline ICI-based therapy than anti-BRAF/MEK therapy, raising questions about the optimal treatment sequencing in NSCLC (16, 30–33). In our study, ICI-based regimens led to survival outcomes that were comparable to anti-BRAF/MEK therapy. This aligns with clinical trial and real-world data reporting a PFS of 10.5 – 18.5 months and OS of 28.0 – 43.3 months for ICI-based therapy, versus PFS of 10.8 – 30.2 months and OS of 17.3 – NE for anti-BRAF/MEK therapy (12, 14, 16, 19, 22, 23, 34–36). In our cohort, we also noticed a clinically meaningful trend favoring OS with ICIs in those with PD-L1 ≥50% (53.1 vs 24.8 months; *p* = 0.61), but the study was underpowered for a definitive conclusion. Patients treated with chemotherapy-alone, on the other hand, experienced significantly worse OS compared to those receiving ICI/chemo-ICI (*p* = 0.03) and trended towards inferior OS compared to anti-BRAF/MEK therapy (*p* = 0.09). While these OS differences could reflect confounding factors from subsequent therapies in each cohort, previous studies reported that chemotherapy has limited efficacy in patients with Class I *BRAF*-mutant NSCLC (1). Taken together, our results suggest that frontline ICIs offer a viable alternative to targeted therapy in individuals with Class I *BRAF*-mutant NSCLC, particularly in those whose PD-L1 expression is elevated. This may be especially relevant given real-world data suggesting that the efficacy of anti-BRAF/MEK therapy may be preserved beyond the frontline setting (37, 38). Randomized studies are needed to confirm these observations, especially given the conflicting data on PD-L1 expression and ICI outcomes for Class I mutations (18, 22, 23).

Individuals with non-Class I mutations present a greater therapeutic challenge. These mutations confer intrinsic resistance to many BRAF-inhibitors as a result of RAS-independent dimerization (4, 39). Consistent with existing literature, no patients with non-Class I mutations in our cohort received anti-BRAF therapy while outcomes with chemotherapy-alone were poor (1). ICI-based therapies, however, improved PFS and trended towards better OS compared to chemotherapy. These findings support current guideline recommendations and expert opinions favoring ICI incorporation for individuals with non-Class I-mutant NSCLC (13, 19, 20, 40–42). Our findings also support the potential of PD-L1 expression as a predictive biomarker of ICI-response in this population (18). For instance, patients treated with ICI-only appeared to outperform those with chemo-ICI, likely reflecting a higher prevalence of PD-L1 ≥50% in those treated with ICI-only. Patients with PD-L1 levels <1% treated with ICI-based therapies also experienced improved survival compared to chemotherapy-alone, suggesting that chemotherapy may have limited – if not detrimental – effects in non-Class I *BRAF* mutations. These observations are speculative and should be interpreted with caution due to the limited sample size.

An intriguing finding was the survival advantage observed in Class I over non-Class I for individuals treated with ICIs. This differs from reports where survival between both groups was either comparable or favored non-Class I mutations (18, 20, 43). We believe several factors contributed to this discrepancy. First, 60% of individuals in our cohort exhibited high PD-L1 levels ≥1%, which contrasts with the 23% seen in the general NSCLC population (44). Further, PD-L1 ≥1% was more common in Class I (96%) compared to non-Class I mutations (64%), and higher PD-L1 levels correlated with improved outcomes. This suggests that *BRAF*-mutant NSCLC, particularly those with Class I and higher PD-L1, may be more responsive to ICIs compared to other oncogene-driven NSCLCs (i.e., *EGFR* or *ALK*) (18, 45–49). Second, non-Class I mutations were frequently associated with *STK11*, *KEAP1*, and *RAS* family gene alterations (*KRAS*, *NRAS*, *HRAS*). These co-mutations have been previously reported in this population, reflect independent RAS activation, and negatively impact ICIs response (50–53). Dual ICI therapy has been proposed as a strategy to mitigate the negative effects of *STK11/KEAP1* mutations and may be a potential therapeutic approach for patients with non-Class I *BRAF* mutations with concurrent *STK11/KEAP1* mutations (54). Third, clinical and demographic differences may have played a role. While our cohort was representative of other North American populations (high proportion of White individuals; no significant association between *BRAF* mutation Class and sex, histology, or stage), patients with Class I mutations were slightly younger than non-Class I (1, 9, 11, 50). This may have positively impacted ICI responses in patients with Class I mutations (55). Additionally, Class I mutations were more common in never-smokers, and never-smokers have better outcomes among NSCLC with targetable mutations (11, 56). Conversely, tobacco use appeared to correlated with improved outcomes in patients with non-Class I mutations treated with ICIs. We hypothesize that high tobacco exposure may have led to an increased PD-L1 expression resulting in enhanced ICI response (57). Finally, CNS involvement was more prevalent in non-Class I patients. This may have negatively impacted outcomes (50, 58).

Our study has several limitations. Its retrospective, single-institution design limits generalizability. The 11-year study period may have introduced confounders resulting from evolving treatment standards. Self-reported data on ethnicity and tobacco use, as well as provider-documented outcomes, may be subject to bias. Subgroup analyses were mainly exploratory given the small sample size and limited interpretation. Finally, the observed differences between ICI-alone and Chemo-ICI were likely influenced by selection bias as patients with more aggressive disease may have preferentially received chemo-ICI (59).

This study also has important clinical implications for patients with newly diagnosed, advanced, *BRAF*-mutant NSCLC. First, ICI-based therapy appears to be a viable frontline option for patients with Class I mutations, particularly those in whom there is concern about the toxicity from targeted therapies or in those whose PD-L1 levels are ≥50%. The prolonged OS observed in this subgroup compared with anti-BRAF/MEK therapy, although not statistically significant, suggests a potential clinically meaningful benefit in long-term outcomes that warrants confirmation in prospective studies. PD-L1 also appeared to be a useful predictive marker in our cohort, but its role remains unclear and warrants validation in larger clinical trials. Second, in individuals with non-Class I mutations, ICI-based therapy should be considered the backbone of treatment given the lack of benefit observed with chemotherapy-alone. Finally, a comprehensive assessment of the genomic landscape is key, since the presence of co-mutations like *STK11* or *KEAP1* may negatively impact ICI response and affect treatment selection (e.g., dual over single ICI regimen).

## 5 Conclusion

Our findings suggest that ICI-based therapies are an effective treatment option for *BRAF*-mutant NSCLC. In patients with Class I mutations, ICIs appear at least comparable to anti-BRAF/MEK therapies and could provide superior outcomes in patients with high PD-L1 expression. Among non-Class I mutations, ICIs are promising but their benefit seems more limited, likely reflecting a lower PD-L1 expression, a more aggressive co-mutational landscape, and increased rates of CNS involvement. Prospective studies should clarify the predictive role of PD-L1 in *BRAF*-mutant NSCLC, directly compare ICIs and anti-BRAF/MEK therapy in Class I mutations and identify optimal strategies for treating non-Class I mutations such as intensified or combination ICI strategies in those with *STK11/KEAP1* co-alterations.

## Data Availability

The raw data supporting the conclusions of this article will be made available by the authors, without undue reservation.
