# Supplementary material for: Outcomes with frontline immune checkpoint inhibitors among individuals with BRAF-mutant non-small cell lung cancer
Source: Front Oncol. 2025 Dec 10;15:1681119. doi: 10.3389/fonc.2025.1681119 (PMC12727587; doi:10.3389/fonc.2025.1681119)
Supplement: Supplementary file 1 [file DataSheet1.docx]

SUPPLEMENTAL MATERIAL

**Table 1S. Genomic Platforms used to Identify BRAF Mutations**

| **Method** | **Class I (n=54)** | **Non-Class I (n=68)** |
| --- | --- | --- |
| **IHC** | 1 (1.9%) | 0 (0%) |
| **Pyrosequencing** | 18 (33.3%) | 1 (1.5%) |
| Moffitt Pyrosequencing |  |  |
| **Genotyping** | 2 (3.7%) | 0 (0%) |
| SnaPshot |  |  |
| **Sequenom** | 1 (1.9%) | 2 (2.9%) |
| MassARRAY |  |  |
| **Next-Generation Sequencing** | 32 (59.2%) | 65 (95.6%) |
| Guardant 360 | 15 (27.8%) | 35 (51.5%) |
| Caris | 0 (0%) | 1 (1.5%) |
| Foundation One (Liquid, CDx) | 7 (13.0%) | 11 (16.2%) |
| Other | 10 (18.5%) | 18 (26.5%) |

Other: Biocept, Moffitt STAR, CancerPlex, NeoGenomics, TruSight

**Table 2S. Specific BRAF Mutations on Next-Generation Sequencing (NGS)**

| **Mutation** | **N (%)** |
| --- | --- |
| **Class I** | 32 (100%) |
| V600E | 32 (100%) |
| **Class II** | 35 (100%) |
| G469X | 18 (51.4%) |
| G469V | 10 (28.6%) |
| G469A | 5 (14.3%) |
| K601E | 5 (14.3%) |
| G469R | 2 (5.7%) |
| G469A + K601E | 1 (2.9%) |
| G464V | 3 (8.6%) |
| ARMC10 fusion | 1 (2.9%) |
| E586K | 1 (2.9%) |
| K601N | 1 (2.9%) |
| L597X | 2 (5.7%) |
| L597Q | 1 (2.9%) |
| L597R | 1 (2.9%) |
| N486_P490del | 1 (2.9%) |
| PACSIN2 fusion | 1 (2.9%) |
| R260H | 1 (2.9%) |
| TMEM178B-fusion | 1 (2.9%) |
| **Class III** | 30 (100%) |
| N581X | 8 (26.7%) |
| N581S | 5 (16.7%) |
| N581I | 2 (6.7%) |
| N581Y | 1 (3.3%) |
| D594X | 8 (26.7%) |
| D594G | 4 (13.3%) |
| D594N | 3 (10%) |
| D594E | 1 (3.3%) |
| G466X | 7 (23.3%) |
| G466A | 3 (10%) |
| G466V | 3 (10%) |
| G466E | 1 (3.3%) |
| G596X | 4 (13.3%) |
| G596R | 3 (10%) |
| G596V | 1 (3.3%) |
| G446V | 1 (3.3%) |
| N660fs | 1 (3.3%) |
| Splice Variant | 1 (3.3%) |

**Table 3S. *EGFR* Co-Mutations**

| Mutation Class | Specific *EGFR* Alteration | Anti-EGFR therapy? | Line of treatment | Best Response |
| --- | --- | --- | --- | --- |
| Class I (n=5) | T790M | No |  |  |
|  | AMP |  |  |  |
|  | D770_N771insG |  |  |  |
|  | exon 20 s768_Asp770dup |  |  |  |
|  | D247N |  |  |  |
| Non-Class I (n=9) | L858R* | Y | 1^st^ and ≥2^nd^ | SD |
|  | ex19del* | Y | 1^st^ and ≥2 | PR |
|  | A647T | No |  |  |
|  | V851I, H621Y |  |  |  |
|  | AMP |  |  |  |
|  | V802I |  |  |  |
|  | A767_V769dup, Exon20ins |  |  |  |
|  | AMP |  |  |  |
|  | AMP |  |  |  |

* These patients were excluded from outcomes analysis because they did not receive anti-BRAF/MEK, immunotherapy, or chemotherapy in the first-line setting.

**Table 4S. Subsequent line of therapy by mutation class**

| Class | Frontline | Subsequent | Time-on-Treatment (mo) | Best Response | Overall Survival (mo) |
| --- | --- | --- | --- | --- | --- |
| I | Anti-BRAF/MEK (n=15) | Anti-BRAF (n=1) | 12 | SD | 16.1 |
|  |  | Chemo-ICI (n= 2) | 24 | PR | 53.2 |
|  |  |  | Unavailable | Unavailable | 38.2 |
|  |  | ICI-Alone (n=1) | 36 | PR | 36.4 |
|  |  | Chemotherapy (n=1) | 18 | Unavailable | 24.8 |
|  |  | None (n=9) |  |  |  |
|  |  | Unknown (n=1) |  |  |  |
|  | ICI/Chemo-ICI (n=14) | Anti-BRAF/MEK (n=6) | 42 | CR | 47.3 |
|  |  |  | 4 | PR | 108.4 |
|  |  |  | 8 | SD | 53.9 |
|  |  |  | 3 | SD | 12.0 |
|  |  |  | 1 | Unavailable | 16.3 |
|  |  |  | 7 | SD | 53.1 |
|  |  | Chemo-ICI (n=1) | 10 | SD | 20.0 |
|  |  | ICI-Alone (n=1) | 1 | PD | 24.6 |
|  |  | Chemotherapy (n=2) | 4 | SD | 42.6 |
|  |  |  | 7 | PD | 39.2 |
|  |  | Other (n=1) | 1 | Unavailable | 11.8 |
|  |  | None (n=3) |  |  |  |
|  | Chemotherapy (n=11) | Anti-BRAF/MEK (n=1) | 24 | PR | 29.8 |
|  |  | ICI-Alone (n=3) | 5 | Unavailable | 32.8 |
|  |  |  | 1 | PD | 22.2 |
|  |  |  | 1 | SD | 10.1 |
|  |  | Chemotherapy (n=3) | 1 | Unavailable | 46.6 |
|  |  |  | 1 | PD | 46.1 |
|  |  |  | 2 | Unavailable | 11.9 |
|  |  | Other (n=1) | 8 | SD | 12.3 |
|  |  | None (n=2) |  |  |  |
|  |  | Unknown (n=1) |  |  |  |
| Non-Class I | ICI/Chemo-ICI  (n=42) | Anti-BRAF/MEK (n=2) | Unavailable | Unavailable | 23.3 |
|  |  |  | 4 | SD | 36.6 |
|  |  | Chemo-ICI (n=1) | 6 | Unavilable | 25.4 |
|  |  | ICI-Alone (n=2) | 2 | PD | 11.9 |
|  |  |  | 18 | PR | 54.1 |
|  |  | Chemotherapy (n=6) | 3 | SD | 11.0 |
|  |  |  | 2 | SD | 62.8 |
|  |  |  | 1 | Unavailable | 8.8 |
|  |  |  | 3 | PD | 23.1 |
|  |  |  | 1 | Unavailable | 7.0 |
|  |  |  | 1 | SD | 10.7 |
|  |  | Other (n=1) | 4 | SD | 73.8 |
|  |  | None (n=28) |  |  |  |
|  |  | Unknown (n=2) |  |  |  |
|  | Chemotherapy  (n=11) | ICI-Alone (n=5) | 1 | Unavailable | 3.9 |
|  |  |  | 1 | PD | 9.9 |
|  |  |  | 5 | SD | 27.7 |
|  |  |  | 1 | PD | 35.2 |
|  |  |  | 1 | PD | 25.7 |
|  |  | Chemotherapy (n=1) | 8 | SD | 29.6 |
|  |  | None (n=4) |  |  |  |
|  |  | Unknown (n=1) |  |  |  |

**Abbreviations:** ICI (Immune checkpoint inhibitor), mo (months) , SD (Stable Disease), PD (progression of disease), PR (Partial Response), SD (Stable Disease)

**Table 5S A. Response Rate by Frontline Therapy**

**
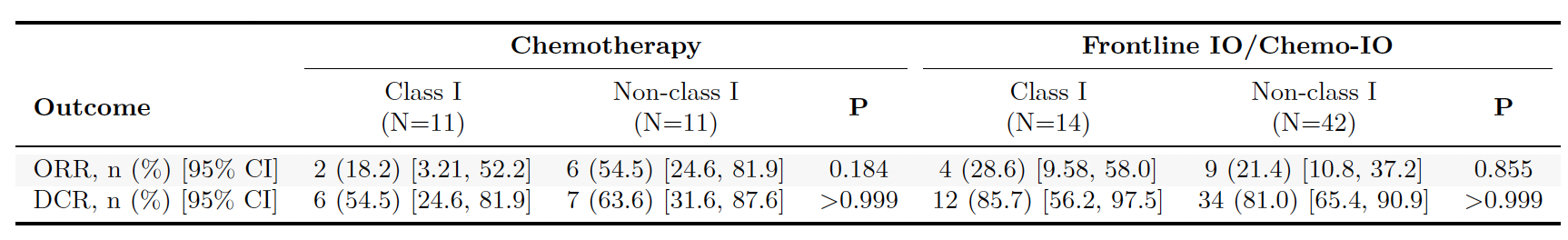
**

**Table 5S B. Response Rate by CNS Involvement**

**
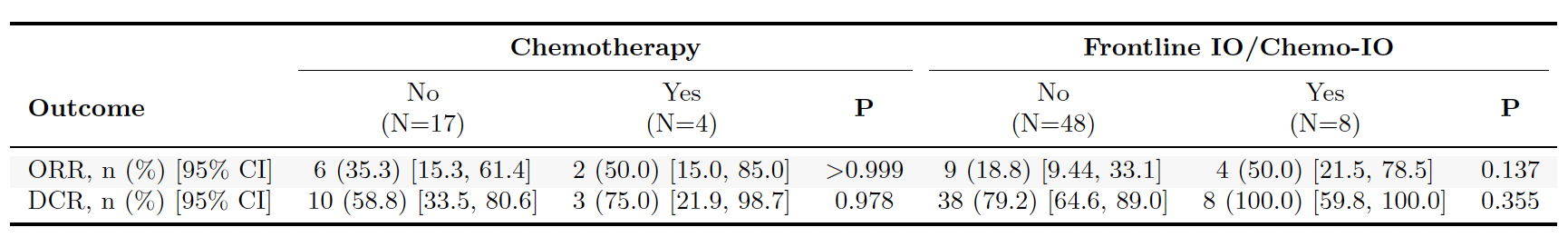
**

**Figure 1S (A). Full OncoMap**

**
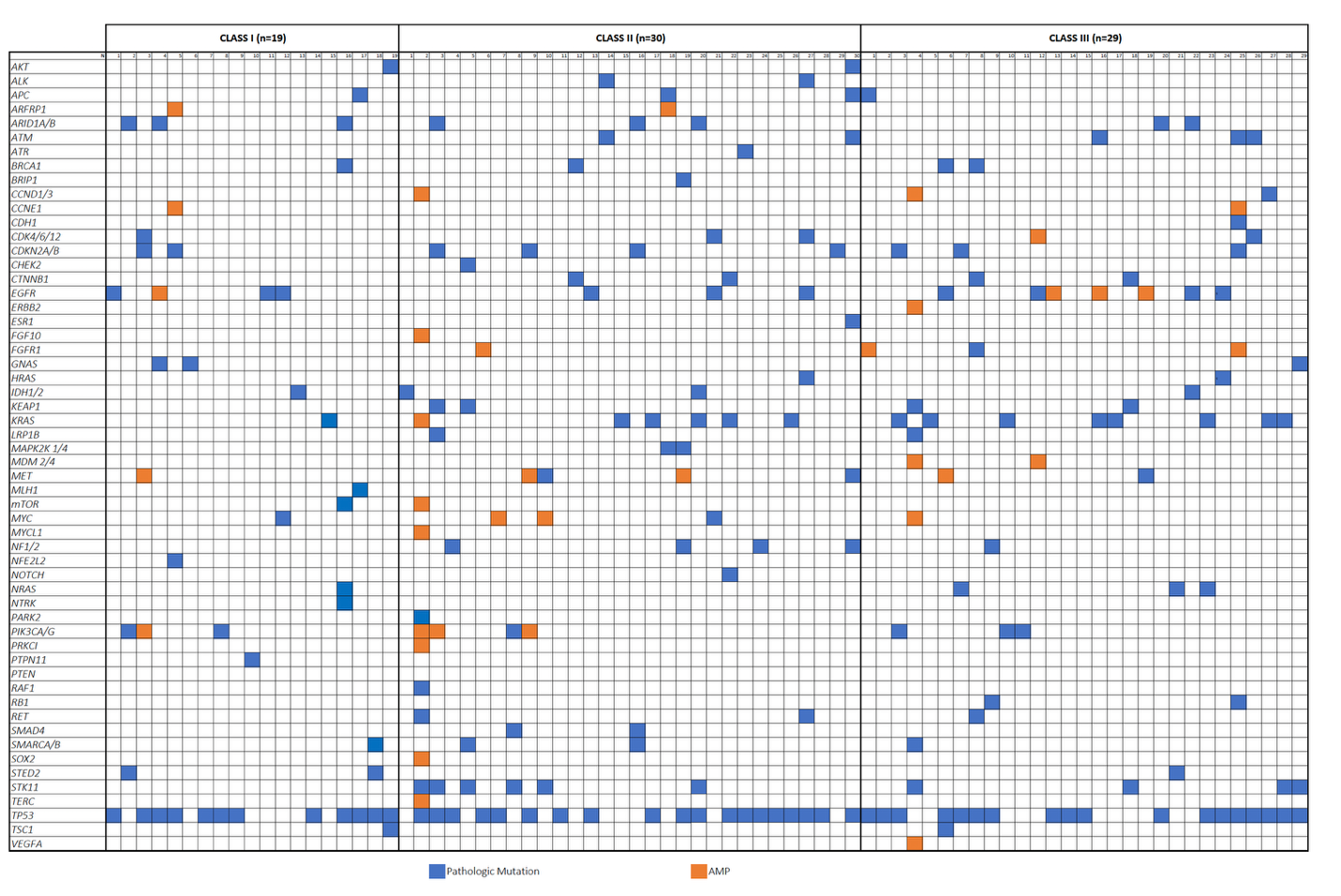
**

**Figure 1S (B). Co-Mutation Distribution**

**Figure 2S. Outcomes with systemic therapy between Class I and non-Class I**

1. **ICI/Chemo-ICI**

**
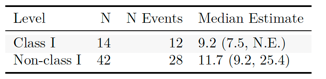

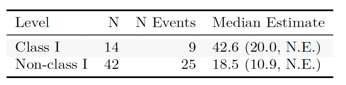

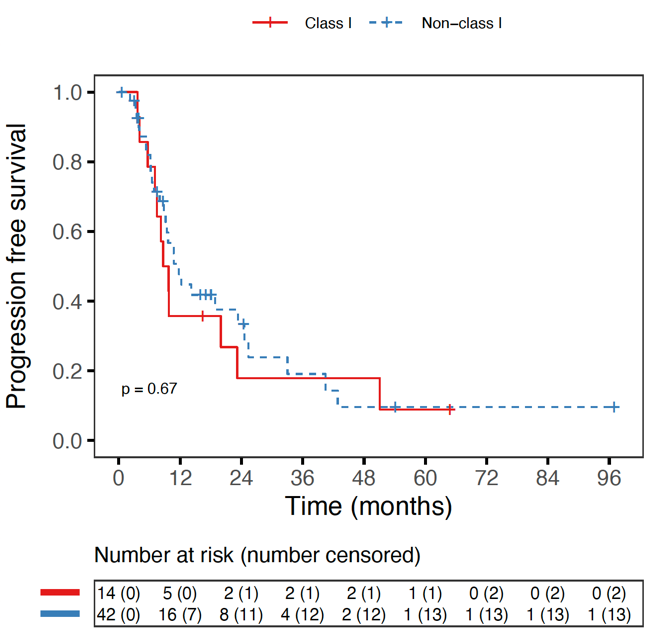

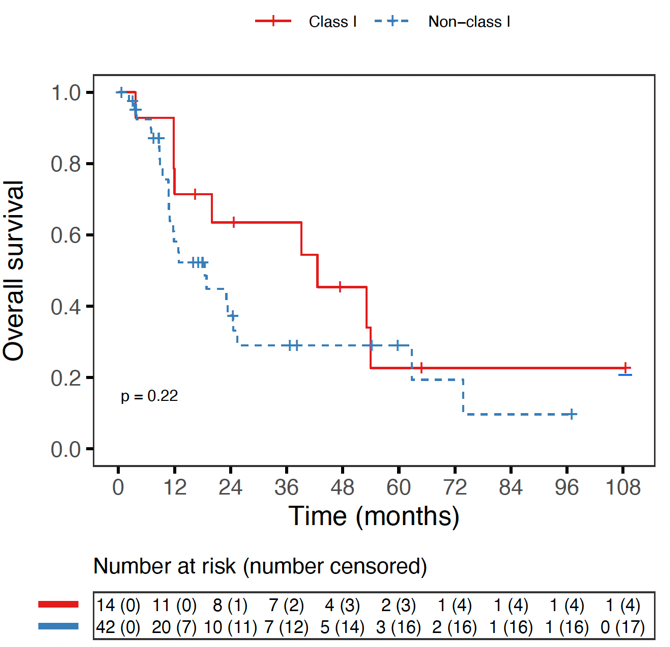
**

1. **Chemotherapy**

**
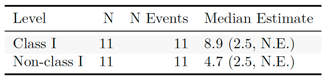

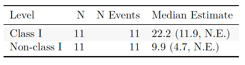

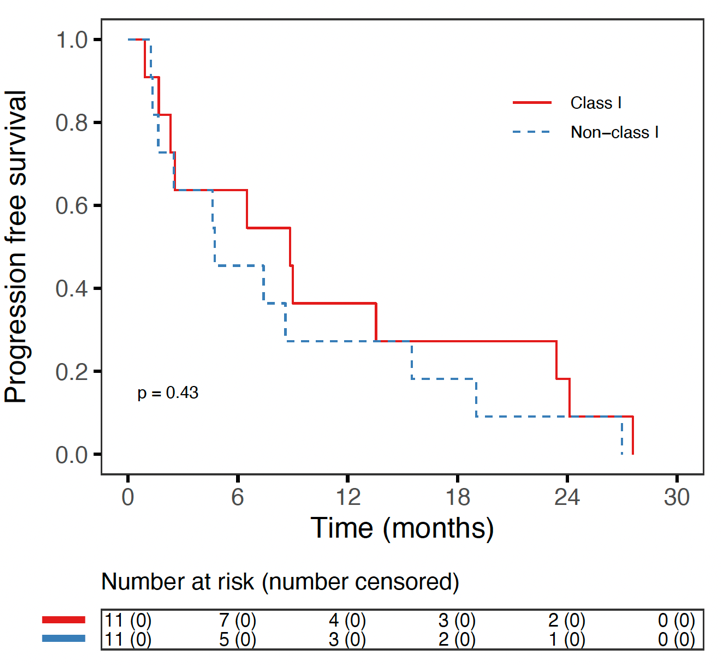

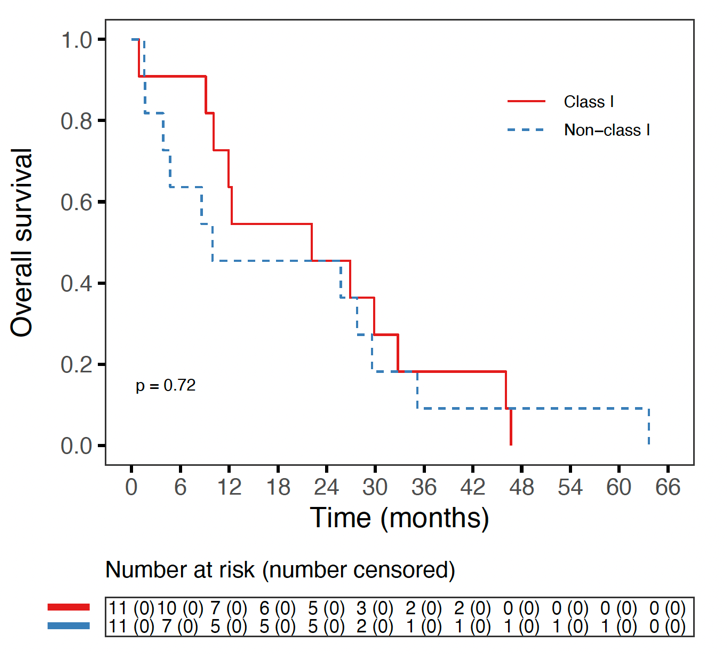
**

**Figure 3S. Outcomes with ICI-Alone or Chemo-ICI Compared to Other Therapies**

1. **ICI-Alone vs. Chemotherapy (All Patients)**

**
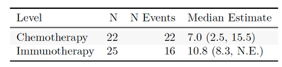

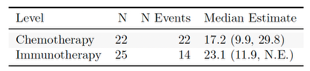

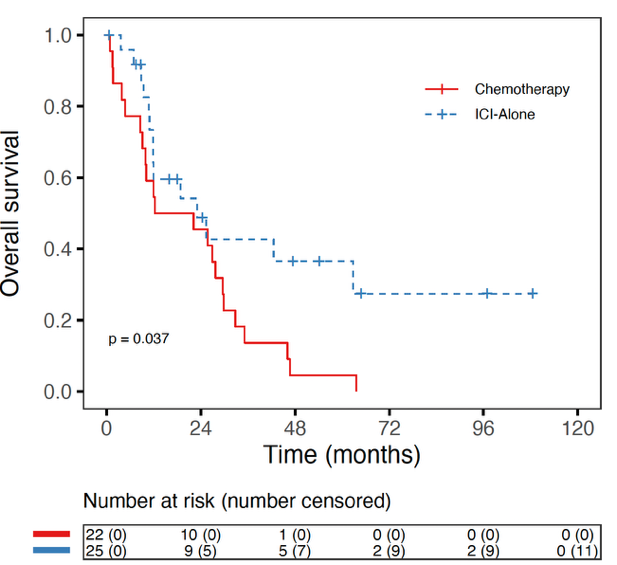

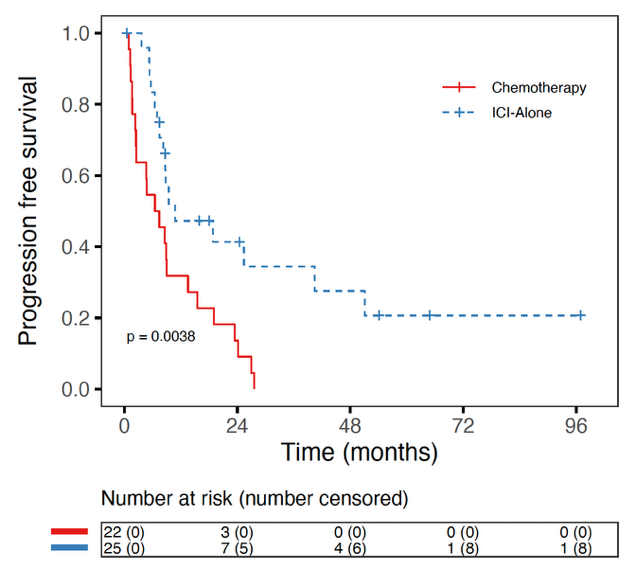
**

1. **Chemo-ICI vs. Chemotherapy (All Patients)**


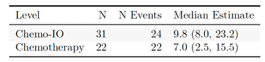

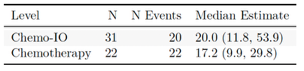

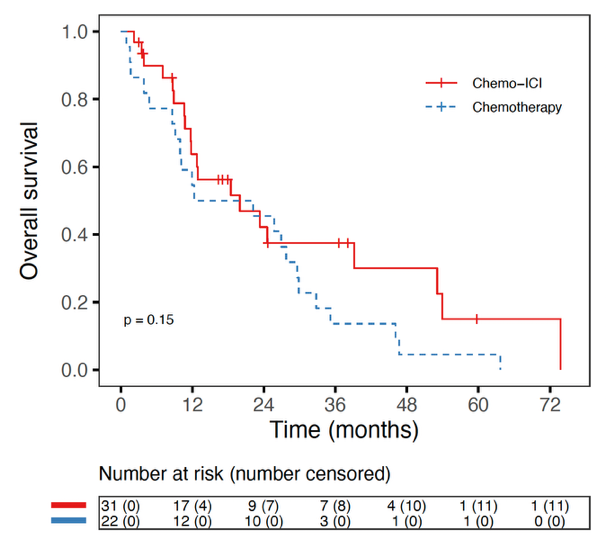

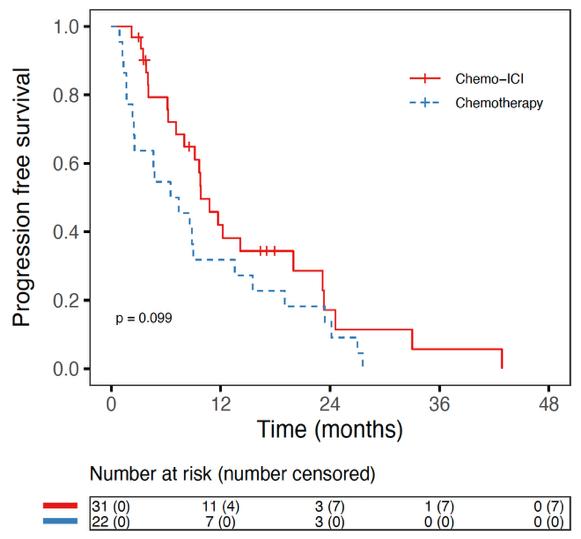


1. **ICI-Alone vs. Chemotherapy (Class I)**

**
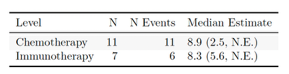

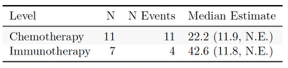

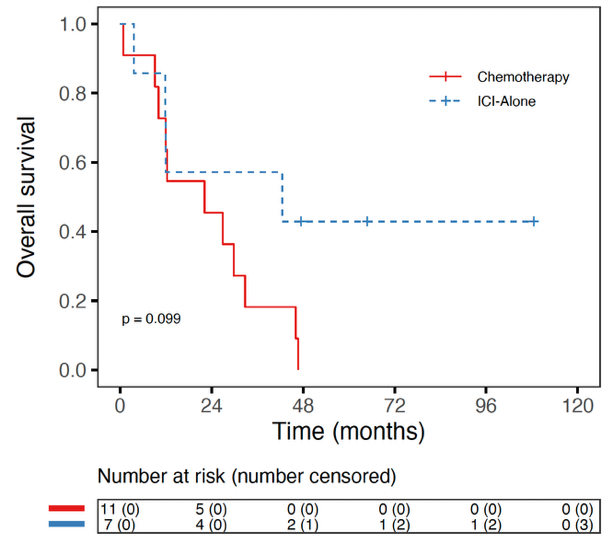

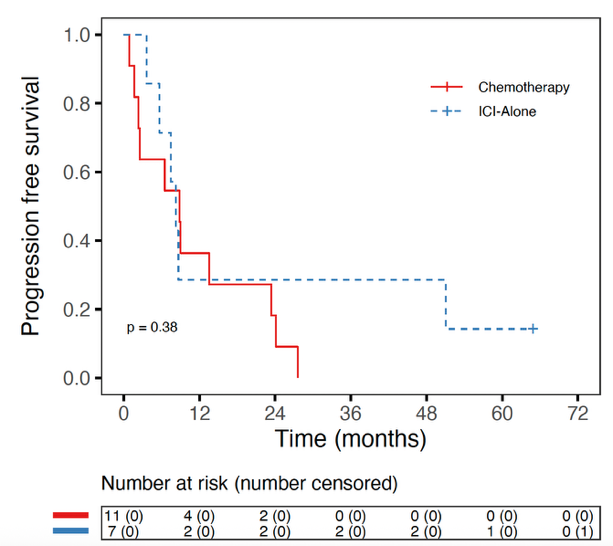
**

1. **Chemo-ICI vs. Chemotherapy (Class I)**


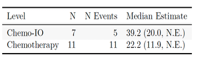

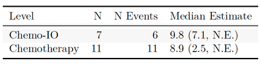

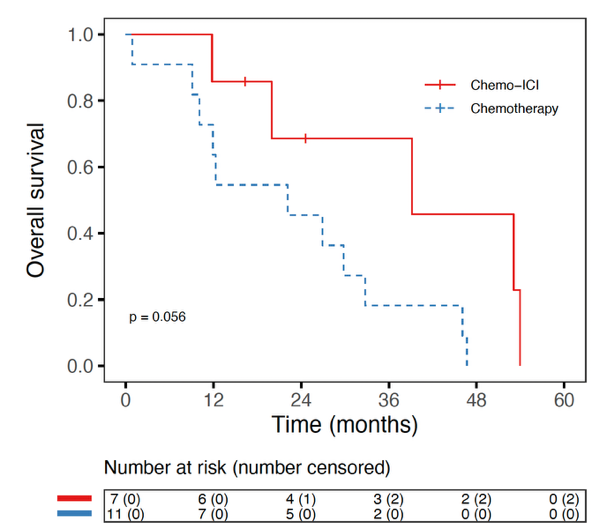

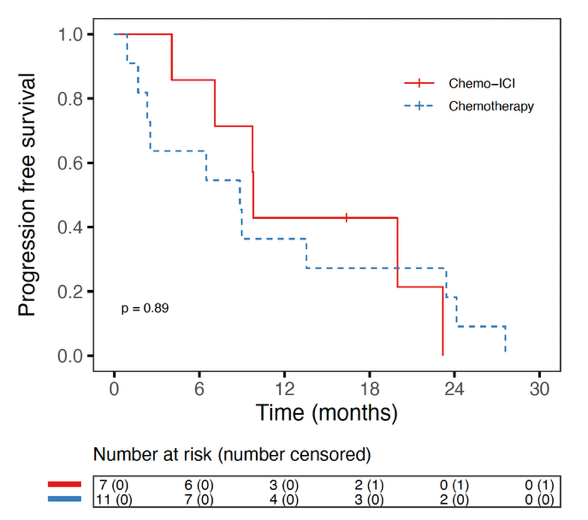


1. **ICI-Alone vs. Anti-BRAF/MEK (Class I)**

**
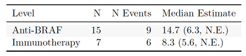

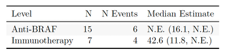

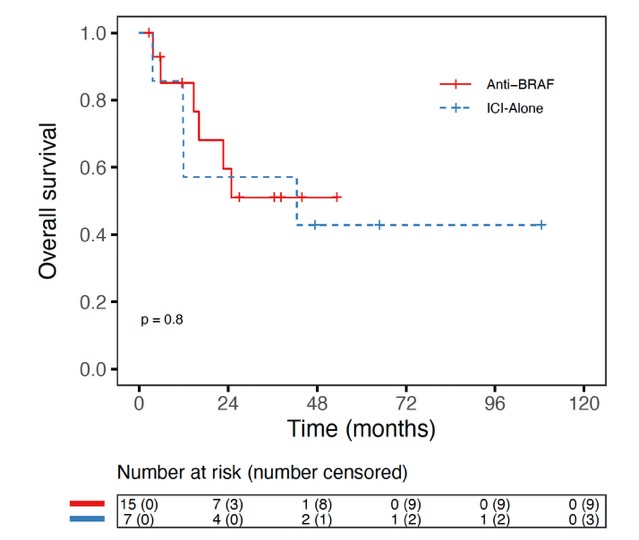

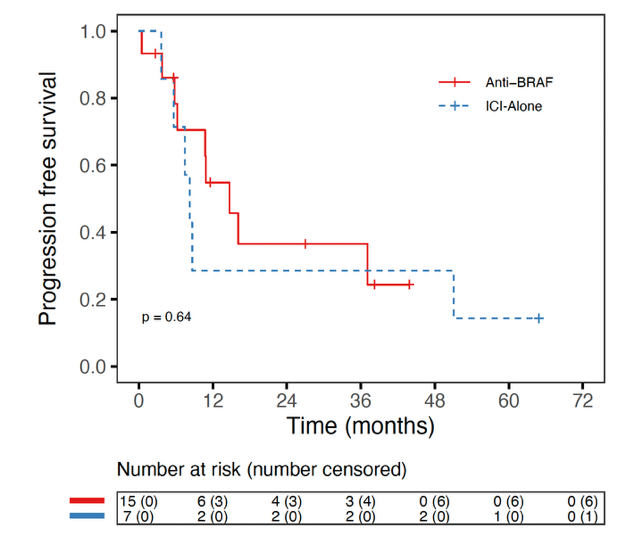
**

1. **Chemo-ICI vs. Anti-BRAF/MEK (Class I)**

**
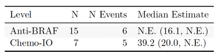

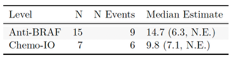

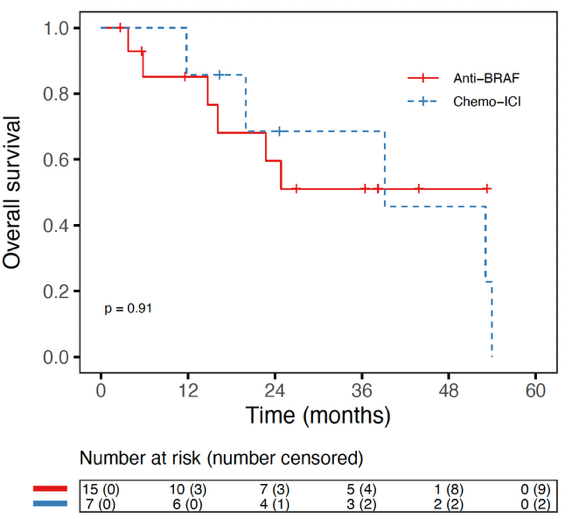

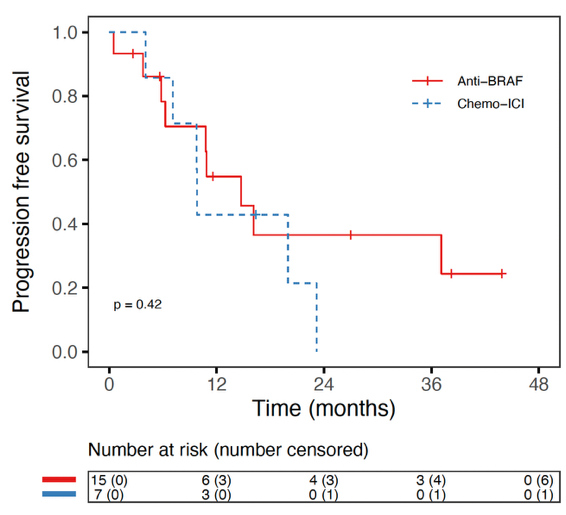
**

1. **ICI-Alone vs. Chemotherapy (Non-Class I)**

**
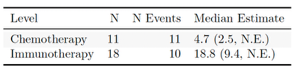

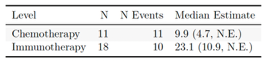

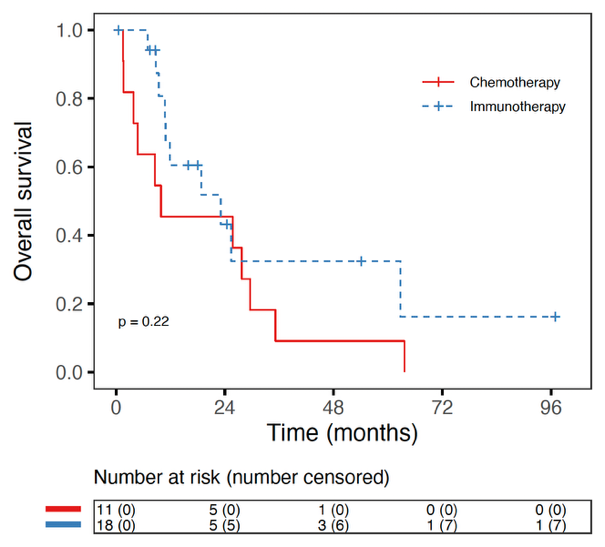

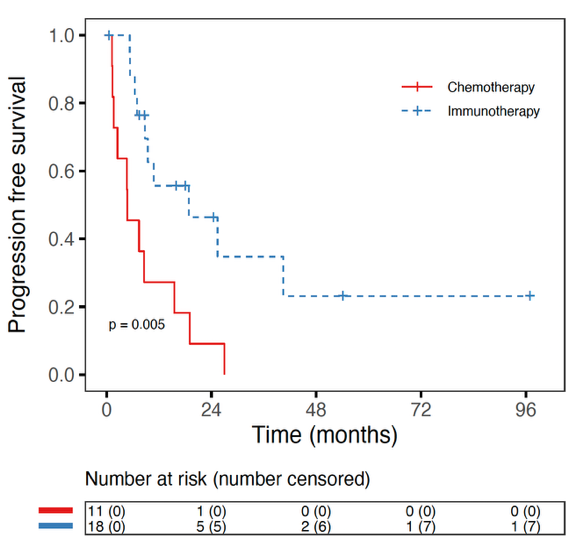
**

1. **Chemo-ICI vs. Chemotherapy (Non-Class I)**


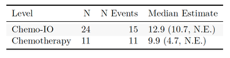
**
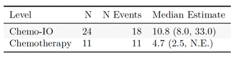
**
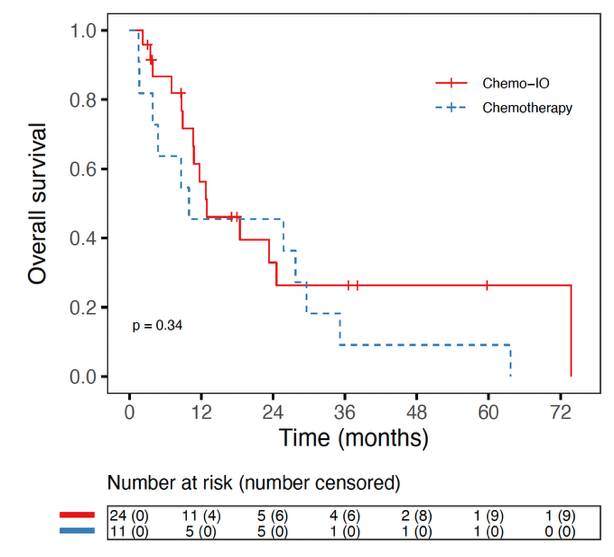
**
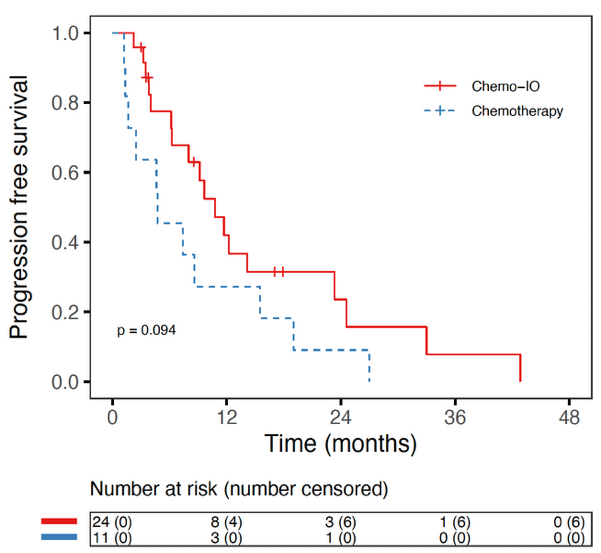
**

**Figure 4S. Outcomes with ICI/Chemo-ICI Depending on Tobacco Use History**

**A. B.**

**
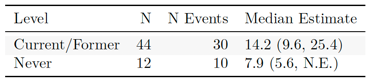

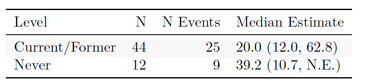

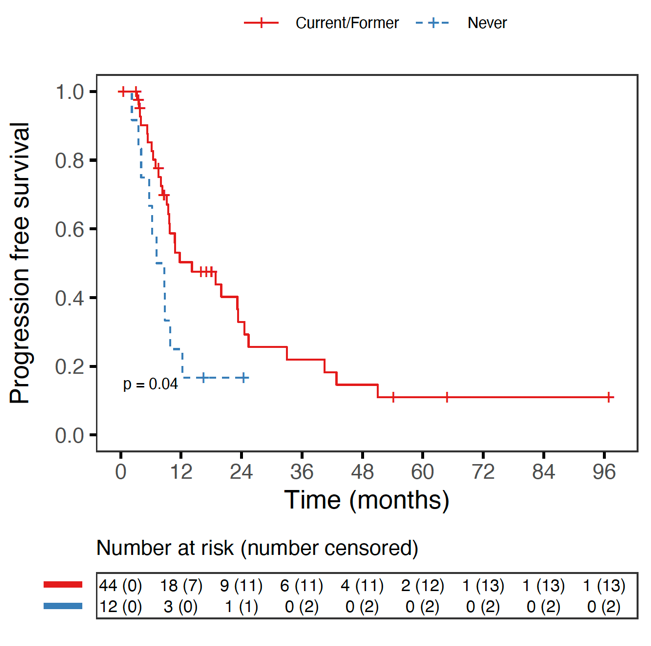

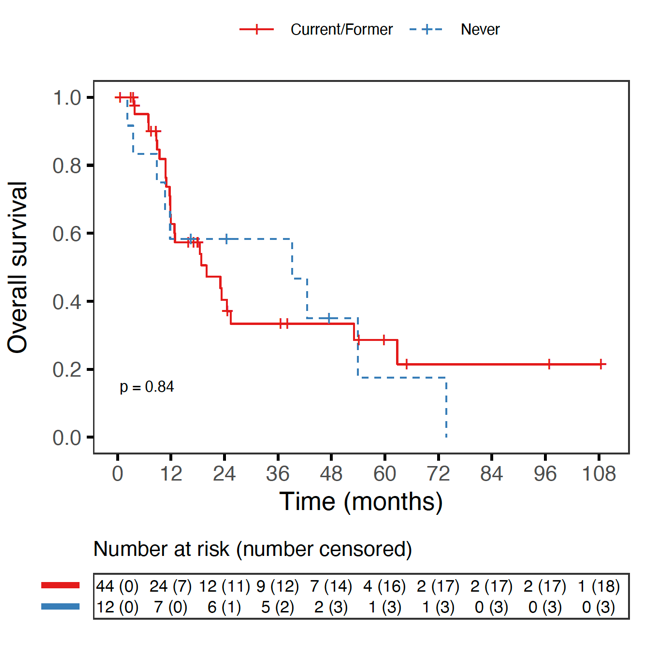
**

**Figure 5S. PFS and OS by CNS involvement**

**A. ICI/Chemo-ICI**

**
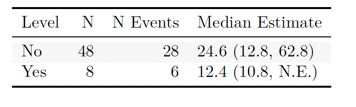

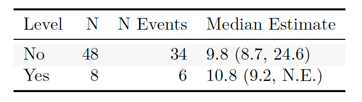

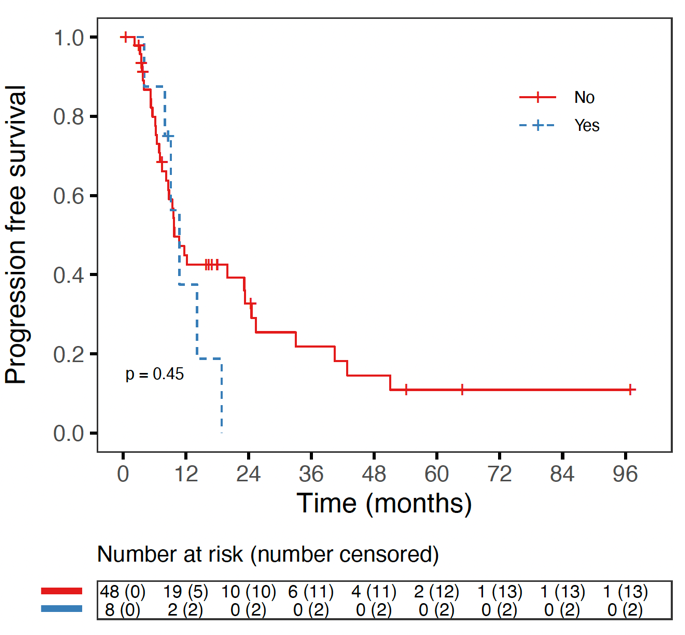

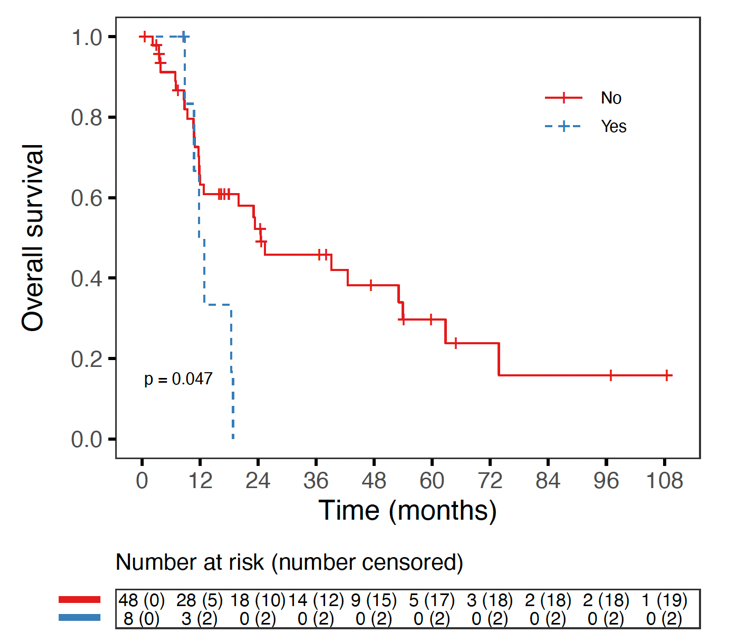
**

**B. Chemotherapy**

**
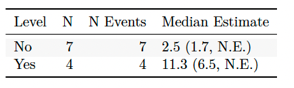

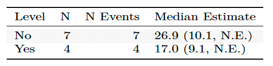

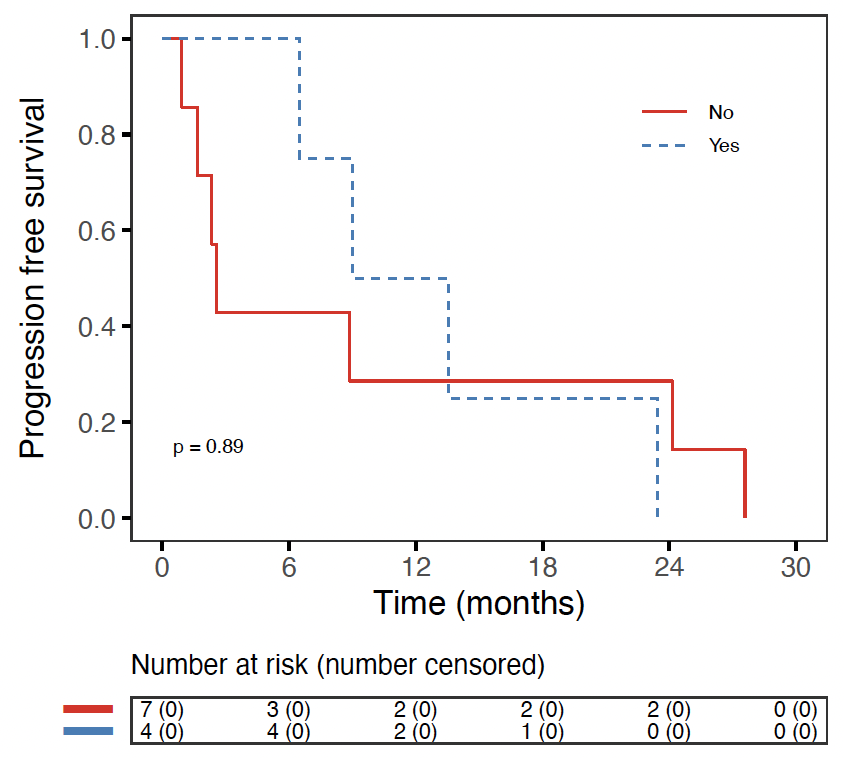

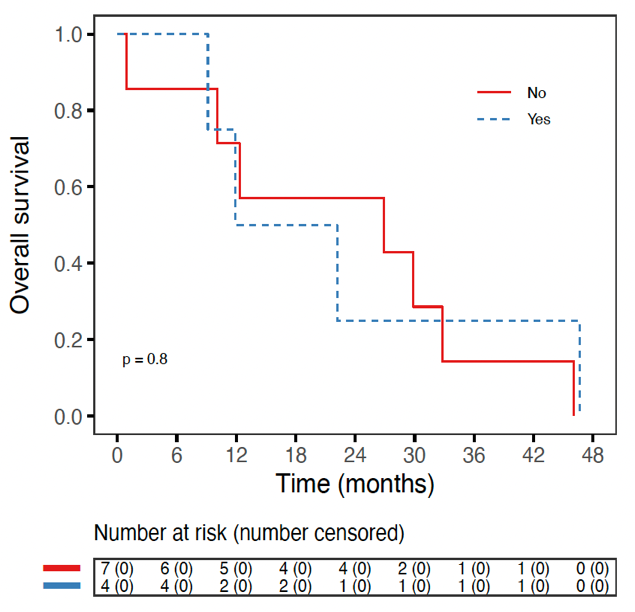
**
