# Supplementary figures and images for: Outcomes with frontline immune checkpoint inhibitors among individuals with BRAF-mutant non-small cell lung cancer
Source: Front Oncol. 2025 Dec 10;15:1681119. doi: 10.3389/fonc.2025.1681119 (PMC12727587; doi:10.3389/fonc.2025.1681119)

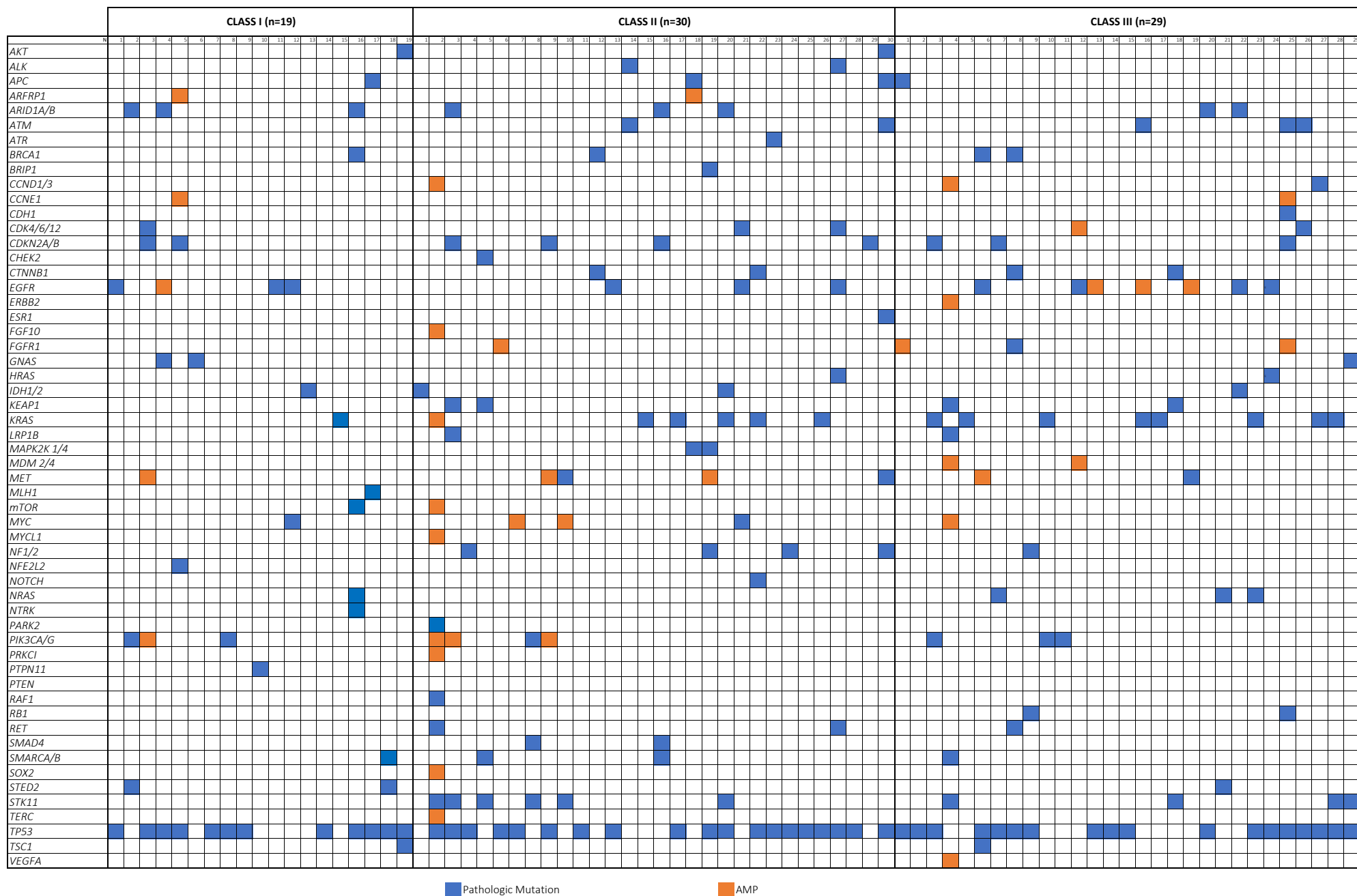

Supplement: Supplementary file 2 [file Supplementaryfile1.pdf]
